# 1^4^,5^4^-Dichloro-3(2,7),7(2,7)-dinaphthal­ena-2,4,6,8-tetra­oxa-1(2,6),5(2,6)-di(1,3,5-triazina)octa­phane

**DOI:** 10.1107/S160053681103460X

**Published:** 2011-08-31

**Authors:** Qiu-Guang Sang, Jing-Kui Yang

**Affiliations:** a College of Chemistry and Chemical Engineering, Graduate University of the Chinese Academy of Sciences, Beijing 100049, People’s Republic of China

## Abstract

In the macrocyclic title compound, C_26_H_12_Cl_2_N_6_O_4_, an O-atom-bridged calix[2]naphthalene­[2]triazine synthesized using a one-pot approach from naphthalene-2,7-diol and cyanuric chloride, the two isolated naphthalene planes and the two triazine-2,6-di­oxy planes adopt a 1,3-alternate configuration, with a dihedral angle of 84.10 (8)° between the naphthalene rings and a dihedral angle of 39.02 (14)° between the triazine rings. In the crystal, weak inter­molecular π–π stacking inter­actions are found between face-to-face naphthalene rings [centroid–centroid distance = 3.662 (7) Å].

## Related literature

For general background and applications of oxocalixarenes, see König & Fonseca (2000 ▶). For background on compounds similar to the title compound and other derivatives from cyanuric chloride reactions, see: Wang & Yang (2004 ▶); Hou *et al.* (2007 ▶); Chen *et al.* (2010 ▶); Zhu *et al.* (2010 ▶); Katz *et al.* (2009 ▶); Katz & Tschaen (2010 ▶); Hu & Chen (2011 ▶).
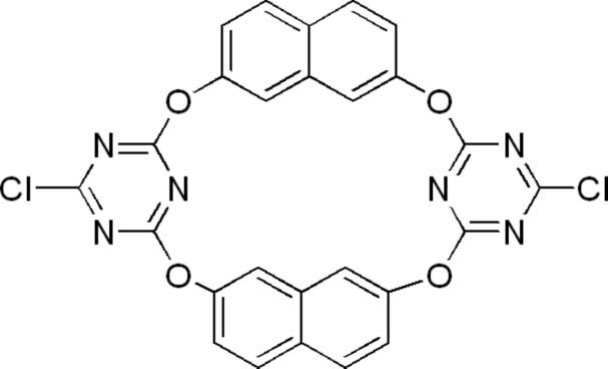


## Experimental

### 

#### Crystal data

C_26_H_12_Cl_2_N_6_O_4_
*M* *_r_* = 543.32
Monoclinic, 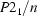

*a* = 15.514 (3) Å
*b* = 7.967 (3) Å
*c* = 18.527 (5) Å
β = 90.60 (2)°
*V* = 2289.8 (11) Å^3^
*Z* = 4
Mo *K*α radiation
μ = 0.33 mm^−1^
*T* = 295 K
0.5 × 0.4 × 0.3 mm

#### Data collection

Bruker P4 diffractometer
5492 measured reflections
4266 independent reflections
2582 reflections with *I* > 2σ(*I*)
*R*_int_ = 0.034
3 standard reflections every 97 reflections  intensity decay: none

#### Refinement

*R*[*F* ^2^ > 2σ(*F* ^2^)] = 0.057
*wR*(*F* ^2^) = 0.123
*S* = 1.03
4266 reflections
343 parameters
H-atom parameters constrained
Δρ_max_ = 0.41 e Å^−3^
Δρ_min_ = −0.39 e Å^−3^

### 

Data collection: *XSCANS* (Bruker, 1997 ▶); cell refinement: *XSCANS*; data reduction: *XSCANS*; program(s) used to solve structure: *SHELXS97* (Sheldrick, 2008 ▶); program(s) used to refine structure: *SHELXL97* (Sheldrick, 2008 ▶); molecular graphics: *SHELXTL* (Sheldrick, 2008 ▶); software used to prepare material for publication: *SHELXTL*.

## Supplementary Material

Crystal structure: contains datablock(s) I, global. DOI: 10.1107/S160053681103460X/zs2134sup1.cif

Structure factors: contains datablock(s) I. DOI: 10.1107/S160053681103460X/zs2134Isup2.hkl

Supplementary material file. DOI: 10.1107/S160053681103460X/zs2134Isup3.cml

Additional supplementary materials:  crystallographic information; 3D view; checkCIF report

## Figures and Tables

**Fig. 1.**
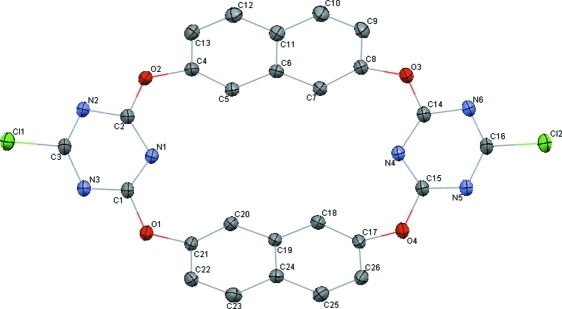
The molecular conformation and atom numbering scheme of the title compound showing showing 50% probability displacement ellipsoids. Hydrogen atoms are omitted.

**Fig. 2.**
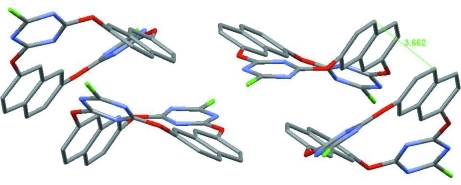
The packing of the title compound showing intermolecular π–π interactions between face-to-face parallel naphthalene rings.

## supplementary crystallographic information

### Comment

Calixarenes and heteroatom-bridged calixaromatics have provided the driving force for the rapid development of supromolecular chemistry (König & Fonseca, 2000). The fast development of miscellaneous oxa-calixarenes may be largely ascribed to the contributions of several research groups (Wang & Yang, 2004; Hou *et al.*, 2007; Zhu *et al.*, 2010; Chen *et al.*, 2010; Katz *et al.*, 2009; Katz & Tschaen, 2010; Hu & Chen, 2011).

In the macrocyclic title compound, C_26_H_12_Cl_2_N_6_O_4_, the oxo-bridged calix[2]naphthalene[2]triazine, which was synthesized using a one-pot procedure from 2,7-naphthalenediol and cyanuric chloride, the molecule adopts a classical 1,3-alternate configuration with the four bridging oxygen atoms located approximately in the same plane (Fig. 1). The distance between two triazine rings varies from 7.006 (12) Å (low rim) to 11.978 (12) Å (upper rim). The distance between two naphthalene rings is 4.048 (12) Å (low rim) or 8.061 (12) Å (upper rim). The dihedral angle between the naphthalene rings is 84.10 (8)° and 39.02 (14)° between the triazine rings. The corresponding angles between triazine rings N1···C2 and N5···C15 and the naphthalene ring C4···C13 are 30.90 (11)° and 27.13 (11)° and to naphthalene ring C17···C26, 64.52 (11)° and 63.57 (11)° respectively. and the inclined angles of the two naphthalene rings are 20.7(x)° and 58.2(x)°, respectively. The length the of C—O bonds between the oxygen bridges and the triazine ring carbon atoms are 1.337(x) Å (C1—O1); 1.332 (3) Å (C2—O2); 1.329 (3) Å (C14—O3) and 1.343 (3) Å (C15—O4), while the oxygen bridges and the naphthalene ring carbon bonds are 1.414 (3) Å (C21—O1); 1.414 (3) Å (C4—O(2); 1.411 (3) Å (C8—O3) and 1.414 (3) Å (C17—O4). This suggests that the oxygen atoms are conjugated with the triazine rings rather than the naphthalene rings.

In the crystal packing of the title compound (Fig. 2) there are relatively short intermolecular interactions involving face-to-face parallel naphthalene rings [ring centroid–centroid separation, 3.662 (7) Å], suggesting weak π-π stacking. In addition there are short intermolecular chlorine···chlorine interactions [Cl1···Cl2^i^, 3.2786 (16) Å] [for symmetry code (i): *x*, *y*, *z* + 1].

### Experimental

To a solution of diisopropylethylamine (DIPEA) (5 mmol, 645 mg) in acetone, 2,7-dihydroxynaphthalene (2 mmol,320 mg) and cyanuric chloride (2 mmol, 369 mg) in acetone were separately but simultaneously added slowly using the high-dilution method. The resulting mixture was then stirred for 24 h until the starting materials were consumed. The solvents were removed, and the residue was chromatographed on a silica gel column to give a pure product (276 mg, yield 51%). Single crystals of the title compound were formed by slow evaporation of a solution in ethyl acetate–petroleum ether.

### Refinement

All H atoms were placed in geometrically calculated positions and refined using a riding model with C—H = 0.93 Å and with  *U*_iso_(H) = 1.2*U*_eq_(C).

### Crystal data

**Table:**

C_26_H_12_Cl_2_N_6_O_4_	*F*(000) = 1104
*M**_r_* = 543.32	*D*_x_ = 1.576 Mg m^−^^3^
Monoclinic, *P*2_1_/*n*	Mo *K*α radiation, λ = 0.71073 Å
Hall symbol: -P 2yn	Cell parameters from 49 reflections
*a* = 15.514 (3) Å	θ = 4.9–12.5°
*b* = 7.967 (3) Å	µ = 0.33 mm^−^^1^
*c* = 18.527 (5) Å	*T* = 295 K
β = 90.60 (2)°	Prism, colorless
*V* = 2289.8 (11) Å^3^	0.5 × 0.4 × 0.3 mm
*Z* = 4	

### Data collection

**Table:**

Bruker P4 diffractometer	*R*_int_ = 0.034
Radiation source: fine-focus sealed tube	θ_max_ = 25.5°, θ_min_ = 2.2°
graphite	*h* = −1→18
ω scans	*k* = −9→1
5492 measured reflections	*l* = −22→22
4266 independent reflections	3 standard reflections every 97 reflections
2582 reflections with *I* > 2σ(*I*)	intensity decay: none

### Refinement

**Table:**

Refinement on *F*^2^	Primary atom site location: structure-invariant direct methods
Least-squares matrix: full	Secondary atom site location: difference Fourier map
*R*[*F*^2^ > 2σ(*F*^2^)] = 0.057	Hydrogen site location: inferred from neighbouring sites
*wR*(*F*^2^) = 0.123	H-atom parameters constrained
*S* = 1.03	*w* = 1/[σ^2^(*F*_o_^2^) + (0.001*P*)^2^ + 2.80*P*] where *P* = (*F*_o_^2^ + 2*F*_c_^2^)/3
4266 reflections	(Δ/σ)_max_ = 0.001
343 parameters	Δρ_max_ = 0.41 e Å^−^^3^
0 restraints	Δρ_min_ = −0.39 e Å^−^^3^

### Special details

**Table:**

Geometry. All e.s.d.'s (except the e.s.d. in the dihedral angle between two l.s. planes) are estimated using the full covariance matrix. The cell e.s.d.'s are taken into account individually in the estimation of e.s.d.'s in distances, angles and torsion angles; correlations between e.s.d.'s in cell parameters are only used when they are defined by crystal symmetry. An approximate (isotropic) treatment of cell e.s.d.'s is used for estimating e.s.d.'s involving l.s. planes.
Refinement. Refinement of *F*^2^ against ALL reflections. The weighted *R*-factor *wR* and goodness of fit *S* are based on *F*^2^, conventional *R*-factors *R* are based on *F*, with *F* set to zero for negative *F*^2^. The threshold expression of *F*^2^ > σ(*F*^2^) is used only for calculating *R*-factors(gt) *etc*. and is not relevant to the choice of reflections for refinement. *R*-factors based on *F*^2^ are statistically about twice as large as those based on *F*, and *R*- factors based on ALL data will be even larger.

### Fractional atomic coordinates and isotropic or equivalent isotropic displacement parameters (Å^2^)

**Table:**

	*x*	*y*	*z*	*U*_iso_*/*U*_eq_	
Cl1	0.65337 (5)	0.52005 (17)	−0.13220 (4)	0.1212 (4)	
Cl2	0.65653 (5)	0.52943 (15)	0.69093 (4)	0.1076 (3)	
O1	0.82098 (10)	0.4316 (3)	0.08191 (8)	0.0752 (6)	
O2	0.53757 (10)	0.2524 (3)	0.07781 (8)	0.0778 (6)	
O3	0.53255 (10)	0.2821 (3)	0.47773 (8)	0.0755 (6)	
O4	0.81654 (11)	0.4587 (3)	0.47411 (8)	0.0830 (7)	
N1	0.68020 (12)	0.3369 (3)	0.08891 (9)	0.0595 (6)	
N2	0.59479 (13)	0.3744 (4)	−0.01723 (10)	0.0766 (8)	
N3	0.73930 (13)	0.4742 (3)	−0.01449 (10)	0.0691 (7)	
N4	0.67511 (12)	0.3658 (3)	0.46679 (9)	0.0602 (6)	
N5	0.73800 (13)	0.4956 (3)	0.57144 (10)	0.0689 (7)	
N6	0.59290 (13)	0.3986 (3)	0.57365 (10)	0.0742 (8)	
C1	0.74257 (15)	0.4122 (4)	0.05242 (11)	0.0617 (8)	
C2	0.60781 (15)	0.3243 (4)	0.05096 (12)	0.0633 (8)	
C3	0.66290 (17)	0.4477 (4)	−0.04477 (12)	0.0747 (10)	
C4	0.52151 (14)	0.1967 (4)	0.14883 (12)	0.0627 (8)	
C5	0.55811 (14)	0.2556 (4)	0.21090 (11)	0.0591 (8)	
H5	0.6042	0.3301	0.2092	0.071*	
C6	0.52496 (13)	0.2016 (3)	0.27861 (11)	0.0538 (7)	
C7	0.55644 (14)	0.2663 (4)	0.34550 (11)	0.0585 (7)	
H7	0.6025	0.3410	0.3467	0.070*	
C8	0.51762 (14)	0.2166 (4)	0.40796 (12)	0.0593 (7)	
C9	0.44956 (16)	0.1016 (4)	0.40900 (13)	0.0718 (9)	
H9	0.4251	0.0699	0.4525	0.086*	
C10	0.41959 (17)	0.0367 (4)	0.34581 (14)	0.0742 (9)	
H10	0.3747	−0.0407	0.3464	0.089*	
C11	0.45556 (15)	0.0848 (4)	0.27896 (13)	0.0596 (7)	
C12	0.42190 (16)	0.0250 (4)	0.21229 (13)	0.0707 (9)	
H12	0.3773	−0.0531	0.2122	0.085*	
C13	0.45381 (16)	0.0801 (4)	0.14867 (13)	0.0680 (8)	
H13	0.4309	0.0409	0.1052	0.082*	
C14	0.60391 (15)	0.3501 (4)	0.50520 (12)	0.0617 (8)	
C15	0.73893 (15)	0.4367 (4)	0.50414 (12)	0.0628 (8)	
C16	0.66226 (16)	0.4676 (4)	0.60166 (12)	0.0711 (9)	
C17	0.83673 (14)	0.3801 (4)	0.40794 (12)	0.0649 (8)	
C18	0.80149 (14)	0.4335 (4)	0.34460 (12)	0.0619 (8)	
H18	0.7573	0.5124	0.3442	0.074*	
C19	0.83321 (13)	0.3669 (3)	0.27905 (11)	0.0526 (7)	
C20	0.80400 (14)	0.4258 (4)	0.21136 (11)	0.0600 (8)	
H20	0.7604	0.5056	0.2083	0.072*	
C21	0.84044 (14)	0.3642 (4)	0.15079 (11)	0.0598 (8)	
C22	0.90542 (15)	0.2455 (4)	0.15168 (12)	0.0688 (9)	
H22	0.9289	0.2073	0.1087	0.083*	
C23	0.93466 (15)	0.1852 (4)	0.21650 (13)	0.0694 (9)	
H23	0.9776	0.1038	0.2176	0.083*	
C24	0.89995 (14)	0.2457 (4)	0.28203 (12)	0.0568 (7)	
C25	0.93288 (16)	0.1943 (4)	0.35013 (13)	0.0692 (8)	
H25	0.9760	0.1133	0.3524	0.083*	
C26	0.90224 (15)	0.2621 (4)	0.41220 (13)	0.0709 (9)	
H26	0.9249	0.2298	0.4567	0.085*	

### Atomic displacement parameters (Å^2^)

**Table:**

	*U*^11^	*U*^22^	*U*^33^	*U*^12^	*U*^13^	*U*^23^
Cl1	0.0746 (4)	0.2384 (12)	0.0506 (3)	0.0012 (6)	−0.0046 (3)	0.0454 (5)
Cl2	0.0756 (4)	0.2001 (10)	0.0471 (3)	0.0050 (6)	0.0059 (3)	−0.0316 (5)
O1	0.0544 (9)	0.1312 (17)	0.0398 (8)	−0.0206 (11)	−0.0037 (7)	0.0095 (10)
O2	0.0486 (8)	0.1420 (18)	0.0427 (8)	−0.0147 (11)	−0.0059 (7)	0.0066 (11)
O3	0.0551 (9)	0.1297 (17)	0.0419 (8)	−0.0138 (11)	0.0075 (7)	−0.0027 (10)
O4	0.0607 (10)	0.1476 (19)	0.0408 (8)	−0.0245 (12)	0.0062 (7)	−0.0147 (11)
N1	0.0488 (10)	0.0919 (17)	0.0378 (9)	−0.0054 (11)	−0.0024 (8)	0.0008 (11)
N2	0.0522 (11)	0.140 (2)	0.0375 (10)	0.0013 (14)	−0.0029 (8)	0.0050 (13)
N3	0.0570 (11)	0.1115 (19)	0.0390 (9)	0.0026 (13)	0.0015 (9)	0.0053 (12)
N4	0.0500 (10)	0.0920 (17)	0.0386 (9)	−0.0036 (11)	0.0027 (8)	0.0032 (11)
N5	0.0604 (11)	0.1070 (18)	0.0392 (9)	0.0003 (13)	0.0023 (9)	−0.0046 (12)
N6	0.0567 (11)	0.125 (2)	0.0406 (10)	0.0021 (14)	0.0079 (9)	−0.0046 (13)
C1	0.0560 (13)	0.093 (2)	0.0364 (11)	−0.0030 (14)	0.0004 (10)	−0.0035 (13)
C2	0.0502 (12)	0.098 (2)	0.0416 (12)	0.0017 (14)	0.0008 (10)	−0.0032 (14)
C3	0.0634 (14)	0.124 (3)	0.0367 (11)	0.0123 (17)	0.0017 (11)	0.0055 (15)
C4	0.0411 (11)	0.102 (2)	0.0450 (12)	0.0043 (14)	0.0000 (10)	0.0044 (14)
C5	0.0420 (11)	0.089 (2)	0.0466 (12)	−0.0048 (13)	−0.0003 (10)	0.0008 (13)
C6	0.0417 (11)	0.0732 (18)	0.0465 (12)	0.0027 (12)	0.0019 (9)	0.0015 (12)
C7	0.0444 (11)	0.085 (2)	0.0456 (12)	−0.0032 (13)	0.0034 (10)	0.0006 (13)
C8	0.0500 (12)	0.0840 (19)	0.0439 (12)	0.0008 (14)	0.0034 (10)	0.0007 (13)
C9	0.0640 (15)	0.100 (2)	0.0515 (13)	−0.0133 (16)	0.0095 (12)	0.0104 (15)
C10	0.0652 (15)	0.094 (2)	0.0636 (15)	−0.0169 (16)	0.0032 (13)	0.0072 (16)
C11	0.0544 (13)	0.0697 (18)	0.0548 (13)	−0.0017 (14)	0.0010 (11)	0.0009 (13)
C12	0.0581 (14)	0.088 (2)	0.0662 (15)	−0.0133 (15)	−0.0057 (12)	0.0018 (16)
C13	0.0553 (13)	0.096 (2)	0.0526 (13)	0.0009 (15)	−0.0088 (11)	−0.0085 (15)
C14	0.0547 (13)	0.089 (2)	0.0414 (12)	0.0026 (14)	0.0035 (10)	0.0065 (13)
C15	0.0557 (13)	0.093 (2)	0.0398 (12)	−0.0008 (15)	0.0043 (10)	0.0062 (13)
C16	0.0646 (15)	0.111 (2)	0.0381 (12)	0.0114 (17)	0.0021 (11)	−0.0040 (14)
C17	0.0473 (12)	0.108 (2)	0.0394 (11)	−0.0155 (15)	0.0051 (10)	−0.0034 (14)
C18	0.0441 (12)	0.094 (2)	0.0478 (12)	−0.0017 (14)	0.0026 (10)	−0.0023 (14)
C19	0.0395 (10)	0.0774 (18)	0.0410 (11)	−0.0060 (12)	0.0017 (9)	0.0009 (12)
C20	0.0449 (12)	0.090 (2)	0.0449 (12)	−0.0001 (13)	−0.0035 (10)	0.0028 (13)
C21	0.0462 (12)	0.095 (2)	0.0385 (11)	−0.0101 (14)	−0.0035 (9)	0.0041 (13)
C22	0.0496 (12)	0.109 (2)	0.0479 (13)	−0.0041 (15)	0.0072 (10)	−0.0134 (15)
C23	0.0493 (13)	0.092 (2)	0.0670 (15)	0.0088 (15)	0.0016 (12)	−0.0102 (16)
C24	0.0423 (11)	0.0809 (19)	0.0471 (12)	−0.0031 (13)	0.0003 (10)	0.0034 (13)
C25	0.0530 (13)	0.094 (2)	0.0600 (14)	0.0030 (15)	−0.0077 (11)	0.0080 (15)
C26	0.0535 (13)	0.111 (2)	0.0477 (13)	−0.0140 (16)	−0.0073 (11)	0.0144 (15)

### Geometric parameters (Å, °)

**Table:**

Cl1—C3	1.724 (2)	C7—C8	1.369 (3)
Cl2—C16	1.729 (2)	C7—H7	0.9300
O1—C1	1.337 (3)	C8—C9	1.398 (4)
O1—C21	1.414 (3)	C9—C10	1.357 (4)
O2—C2	1.332 (3)	C9—H9	0.9300
O2—C4	1.413 (3)	C10—C11	1.417 (3)
O3—C14	1.329 (3)	C10—H10	0.9300
O3—C8	1.411 (3)	C11—C12	1.418 (3)
O4—C15	1.343 (3)	C12—C13	1.356 (4)
O4—C17	1.414 (3)	C12—H12	0.9300
N1—C2	1.323 (3)	C13—H13	0.9300
N1—C1	1.329 (3)	C17—C18	1.358 (3)
N2—C3	1.315 (3)	C17—C26	1.386 (4)
N2—C2	1.338 (3)	C18—C19	1.418 (3)
N3—C3	1.323 (3)	C18—H18	0.9300
N3—C1	1.335 (3)	C19—C20	1.409 (3)
N4—C14	1.326 (3)	C19—C24	1.416 (3)
N4—C15	1.328 (3)	C20—C21	1.354 (3)
N5—C16	1.326 (3)	C20—H20	0.9300
N5—C15	1.333 (3)	C21—C22	1.382 (4)
N6—C16	1.311 (3)	C22—C23	1.367 (3)
N6—C14	1.338 (3)	C22—H22	0.9300
C4—C5	1.361 (3)	C23—C24	1.418 (3)
C4—C13	1.402 (4)	C23—H23	0.9300
C5—C6	1.427 (3)	C24—C25	1.417 (3)
C5—H5	0.9300	C25—C26	1.361 (4)
C6—C11	1.424 (3)	C25—H25	0.9300
C6—C7	1.424 (3)	C26—H26	0.9300
			
C1—O1—C21	120.63 (19)	C13—C12—H12	119.6
C2—O2—C4	129.45 (18)	C11—C12—H12	119.6
C14—O3—C8	129.02 (18)	C12—C13—C4	119.5 (2)
C15—O4—C17	120.6 (2)	C12—C13—H13	120.2
C2—N1—C1	112.49 (19)	C4—C13—H13	120.2
C3—N2—C2	112.6 (2)	N4—C14—O3	121.9 (2)
C3—N3—C1	111.1 (2)	N4—C14—N6	126.6 (2)
C14—N4—C15	112.40 (19)	O3—C14—N6	111.5 (2)
C16—N5—C15	110.8 (2)	N4—C15—N5	128.5 (2)
C16—N6—C14	112.6 (2)	N4—C15—O4	120.4 (2)
N1—C1—N3	128.2 (2)	N5—C15—O4	111.1 (2)
N1—C1—O1	120.5 (2)	N6—C16—N5	129.1 (2)
N3—C1—O1	111.2 (2)	N6—C16—Cl2	116.61 (18)
N1—C2—O2	121.8 (2)	N5—C16—Cl2	114.27 (19)
N1—C2—N2	126.7 (2)	C18—C17—C26	123.4 (2)
O2—C2—N2	111.4 (2)	C18—C17—O4	121.3 (3)
N2—C3—N3	128.8 (2)	C26—C17—O4	114.8 (2)
N2—C3—Cl1	116.84 (19)	C17—C18—C19	118.9 (2)
N3—C3—Cl1	114.4 (2)	C17—C18—H18	120.6
C5—C4—C13	122.4 (2)	C19—C18—H18	120.6
C5—C4—O2	127.1 (2)	C20—C19—C24	119.3 (2)
C13—C4—O2	110.2 (2)	C20—C19—C18	121.8 (2)
C4—C5—C6	119.2 (2)	C24—C19—C18	118.8 (2)
C4—C5—H5	120.4	C21—C20—C19	118.9 (2)
C6—C5—H5	120.4	C21—C20—H20	120.5
C11—C6—C7	119.0 (2)	C19—C20—H20	120.5
C11—C6—C5	118.7 (2)	C20—C21—C22	123.3 (2)
C7—C6—C5	122.2 (2)	C20—C21—O1	121.6 (2)
C8—C7—C6	118.8 (2)	C22—C21—O1	114.8 (2)
C8—C7—H7	120.6	C23—C22—C21	119.1 (2)
C6—C7—H7	120.6	C23—C22—H22	120.4
C7—C8—C9	122.7 (2)	C21—C22—H22	120.4
C7—C8—O3	126.7 (2)	C22—C23—C24	120.5 (3)
C9—C8—O3	110.3 (2)	C22—C23—H23	119.8
C10—C9—C8	119.3 (2)	C24—C23—H23	119.8
C10—C9—H9	120.3	C19—C24—C25	119.2 (2)
C8—C9—H9	120.3	C19—C24—C23	118.9 (2)
C9—C10—C11	121.1 (3)	C25—C24—C23	121.8 (2)
C9—C10—H10	119.5	C26—C25—C24	120.8 (3)
C11—C10—H10	119.5	C26—C25—H25	119.6
C10—C11—C12	121.7 (2)	C24—C25—H25	119.6
C10—C11—C6	119.1 (2)	C25—C26—C17	118.9 (2)
C12—C11—C6	119.2 (2)	C25—C26—H26	120.5
C13—C12—C11	120.9 (3)	C17—C26—H26	120.5
